# Contrasting Immunopathogenic and Therapeutic Roles of Granulocyte Colony-Stimulating Factor in Cancer

**DOI:** 10.3390/ph13110406

**Published:** 2020-11-20

**Authors:** Annette J. Theron, Helen C. Steel, Bernardo L. Rapoport, Ronald Anderson

**Affiliations:** 1 Department of Immunology, Faculty of Health Sciences, University of Pretoria, Pretoria 0001, South Africa; helen.steel@up.ac.za (H.C.S.); bernardo.rapoport@up.ac.za (B.L.R.); ronald.anderson@up.ac.za (R.A.); 2 The Medical Oncology Centre of Rosebank, Johannesburg 2196, South Africa

**Keywords:** cancer, febrile neutropenia, immunosuppression, myeloid-derived suppressor cells, neutrophils, neutrophil extracellular traps (NETs), recombinant granulocyte colony-stimulating factor, regulatory T cells, T helper 2 cells

## Abstract

Tumor cells are particularly adept at exploiting the immunosuppressive potential of neutrophils as a strategy to achieve uncontrolled proliferation and spread. Recruitment of neutrophils, particularly those of an immature phenotype, known as granulocytic myeloid-derived suppressor cells, is achieved via the production of tumor-derived granulocyte colony-stimulating factor (G-CSF) and neutrophil-selective chemokines. This is not the only mechanism by which G-CSF contributes to tumor-mediated immunosuppression. In this context, the G-CSF receptor is expressed on various cells of the adaptive and innate immune systems and is associated with induction of T cell polarization towards the Th2 and regulatory T cell (Treg) phenotypes. In contrast to the potentially adverse effects of sustained, endogenous production of G-CSF by tumor cells, stringently controlled prophylactic administration of recombinant (r) G-CSF is now a widely practiced strategy in medical oncology to prevent, and in some cases treat, chemotherapy-induced severe neutropenia. Following an overview of the synthesis, structure and function of G-CSF and its receptor, the remainder of this review is focused on: (i) effects of G-CSF on the cells of the adaptive and innate immune systems; (ii) mechanisms by which this cytokine promotes tumor progression and invasion; and (iii) current clinical applications and potential risks of the use of rG-CSF in medical oncology.

## 1. Introduction

Colony-stimulating factors (CSFs) are hematopoietic growth factors that are released in response to infection or inflammation to stimulate hematopoietic stem cells to proliferate and generate colonies of differentiated progeny such as neutrophils or macrophages. There are four distinct types of CSFs, each with specific types of receptors, with the occurrence of cross-talk between these receptors, if any, being largely unknown. The CSFs are named after the major types of colonies that they initiate, specifically, granulocyte colony-stimulating factor (G-CSF), granulocyte-macrophage colony-stimulating factor (GM-CSF), macrophage colony-stimulating factor (M-CSF) and multipotential colony-stimulating factor (most commonly termed interleukin-3) [1].

G-CSF, the focus of the current review, stimulates the proliferation and differentiation of neutrophil precursors, to maintain the number of circulating mature and functional neutrophils.

In the therapeutic setting, major applications of administration of recombinant (r) G-CSF include: (i) attenuation of the magnitude and duration of chemotherapy-induced neutropenia in cancer patients [2], described in greater detail below; (ii) treatment of cyclic and chronic neutropenias [3,4]; and (iii) mobilization of hematopoietic progenitor cells into peripheral blood to be harvested for stem cell transplantation. In the latter setting, the incidence and severity of experimental acute graft-versus-host disease (aGVHD) are also reduced by administration of rG-CSF [5,6], seemingly due to the immunoregulatory effects of rG-CSF on cells of both the innate and adaptive immune systems, especially T cells, as reviewed earlier by Yang et al. [7], as well as immature neutrophils [7,8]. The current review builds on these earlier studies and also addresses the origins and structure of the growth factor and its receptor. Other topics covered include the involvement of tumor-derived G-CSF in tumorigenesis, as well as the therapeutic applications of rG-CSF in medical oncology, in addition to the associated potential risks.

## 2. Synthesis and Function of G-CSF

**Figure 1:**
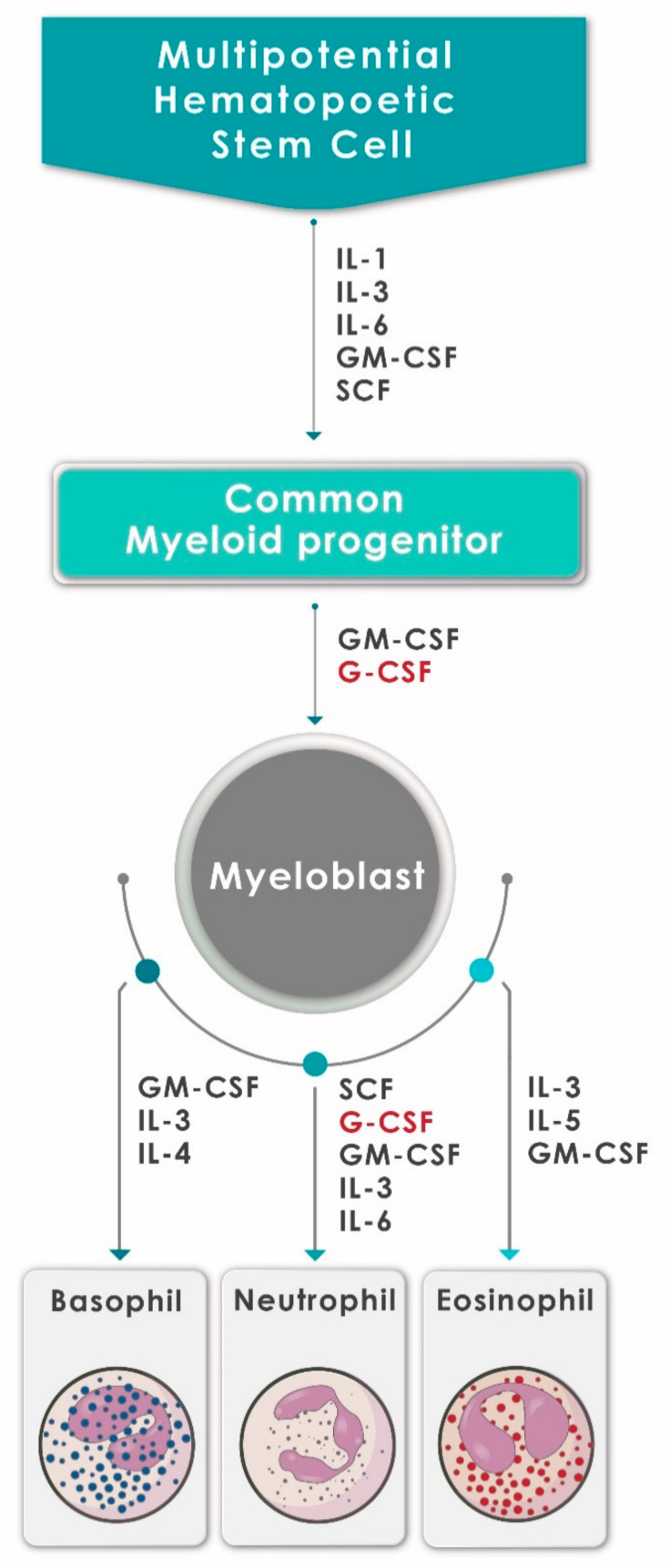
A schematic diagram showing the generation of neutrophils (and other granulocytes) from hematopoietic progenitor cells under the influence of various growth factors and cytokines, including G-CSF (adapted and reproduced from Mehta et al., J Immunol 2015;195:1341–1349; Copyright 2018. The American Association of Immunologists, Inc., Rockville, MD, USA).

Neutrophils are continuously produced from bone marrow-derived pluripotent hematopoietic stem cells via the process of granulopoiesis in which G-CSF plays a key role not only by regulating the production of neutrophils, but also the activity of these cells [9,10]. G-CSF stimulates the proliferation of hematopoietic progenitors by accelerating their cell cycle rate, thus reducing the transit time through granulopoiesis, via enhancement of the transition of immature metamyelocytes into mature neutrophils and acceleration of their release into the circulation [10]. Apart from mediating the survival of neutrophils and their precursors, G-CSF also promotes the antimicrobial functions of mature neutrophils, such as phagocytosis, superoxide production and pathogen killing [11]. The generation of neutrophils (and other types of granulocytes) from hematopoietic progenitor cells under the influence of various growth factors and cytokines, including G-CSF, is depicted in Figure 1.

Endogenous production of G-CSF is largely triggered by infection and tissue damage in response to production of several pro-inflammatory stimuli such as the cytokines interleukin-1 beta (IL-1β) and tumor necrosis factor-alpha (TNFα), as well as bacterial lipopolysaccharides (LPS) [12,13]. G-CSF is found in a wide variety of tissue types, but is mainly produced by resting or stimulated stromal cells of the hematopoietic microenvironment (fibroblasts and endothelial cells) and by cells of the innate immune system such as monocytes and macrophages [14].

The importance of G-CSF in the regulation of granulopoiesis became clear following the findings of two early studies based on mice deficient in expression of either G-CSF or the G-CSF receptor (G-CSFR). These mice displayed chronic neutropenia with a corresponding decrease in granulocytic precursors in the bone marrow [15,16]. Despite these abnormalities, mature neutrophils remained detectable, albeit in much lower numbers, in the blood and bone marrow, indicating the existence of both G-CSF-dependent and -independent mechanisms of granulopoiesis [15,16], probably involving GM-CSF, IL-3 and IL-6.

According to Toghraie et al. (2019), four different mRNA isoforms resulting from alternative splicing have been reported for G-CSF (transcript variants 1, 2, 3 and 4) [17]. Transcript variants 1 and 2 encode the mature 177-amino acid G-CSF isoform A and the 174-amino acid G-CSF isoform B, respectively [17]. Isoform B is the major isoform produced in prokaryotic and eukaryotic expression systems and has more potent biological activity and greater stability than isoform A [17,18]. The other recently described G-CSF isoforms are the 141-amino acid isoform C and the 138-amino acid isoform D that are encoded by transcript variants 3 and 4, respectively [17], the activities of which remain to be established.

## 3. The G-CSF Receptor

The biological activities of G-CSF are mediated via the G-CSFR, which belongs to the class I cytokine receptor superfamily [13]. This receptor is expressed on a range of hematopoietic cells. These include mature neutrophilic granulocytes, myeloid progenitors, hematopoietic stem cells, monocytes and lymphocytes, as well as certain non-hematopoietic tissues [19].

The G-CSFR is a single-chain membrane protein composed of extracellular, transmembrane and intracellular regions [10,19]. The large glycosylated extracellular region contains three distinct functional zones: an N-terminal immunoglobulin (Ig)-like domain, a cytokine receptor homology (CRH) domain and three fibronectin (FBN) type III domains [13]. The CRH domain has four highly conserved cysteines and a Trp-Ser-X-Trp-Ser (WSXWS) motif that are required for ligand recognition and receptor activation [13,20].

The intracellular region of the G-CSFR comprises three distinct box regions. Boxes 1 and 2 are required for activation of proliferation signals, while box 3 promotes the differentiation of myeloid progenitor cells [20]. In addition, phosphorylation of the four tyrosine residues at positions 704, 729, 744 and 764 is also a key event in intracellular signaling, resulting from phosphorylation of multiple SH2 (Src Homology 2)-containing signaling moieties [10,20].

G-CSF binds to the extracellular domains of the G-CSFR, triggering receptor homodimerization, leading to the activation of intracellular signaling cascades involving the Janus kinase-signal transduction and activator of transcription (JAK/STAT), phosphatidylinositol 3-kinase/protein kinase B (PI3K/AKT) and mitogen-activated protein kinase/extracellular signal-regulated kinase 1/2 (MAPK/ERK); these signaling pathways initiate the transcriptional changes that promote proliferation differentiation, migration and survival [19,20,21].

According to Mehta et al. (2014), seven mRNA isoforms encoding the G-CSFR have been isolated with only class 1 (the canonical type) and class IV (differentiation-defective) detectable in hematopoietic cells [22].

## 4. Effects of rG-CSF on Cells of the Adaptive Immune System

Recombinant G-CSF appears to affect T cell homeostasis by multiple mechanisms. Most importantly, these include induction of T cell polarization toward the T helper 2 (Th2) phenotype, as well as inhibition of T cell proliferation, and enhancement of T cell apoptosis. This cytokine may affect T cells either directly or indirectly, by regulating the activities of various cell types such as dendritic cells, monocytes, myeloid-derived suppressor cells (MDSCs) and others [7].

### 4.1. T Cell Proliferation

The proliferative responses of T lymphocytes to both allogeneic and mitogenic stimulation are profoundly reduced in patients, as well as healthy stem cell donors, following treatment with rG-CSF [23,24,25]. These responses normalized 14 days after administration of the last dose of rG-CSF [24]. The inhibitory effects of rG-CSF on T cell proliferation were achieved, at least in part, by an indirect mechanism. This contention is based on observations that soluble factors present in serum from healthy donors who had received rG-CSF also inhibited T cell proliferation to mitotic challenge, possibly due to perturbation of lymphocyte mitochondrial function and inhibition of cell cycle progression [26,27]. Other studies have suggested that administration of rG-CSF suppresses T cell proliferative responses indirectly by modulating monocyte function, as depletion of monocytes in the cell co-culture partially reversed the abnormal proliferative capacity of T cells [23,28].

### 4.2. T Helper 2 Cell Polarization

As alluded to above, rG-CSF may polarize the transition of T cells from the Th1 to Th2 phenotype by switching the cellular cytokine secretion profile. In this context, several studies reported that both murine and human T cells pre-treated with rG-CSF followed by stimulation with mitogens or alloantigens in vitro demonstrated significant decreases in the production of the Th1 cytokines interferon-gamma (IFNγ) and interleukin (IL)-2, while expression of the Th2 cytokines IL-4 and IL-10 was increased [5,29,30,31]. Another study observed that polarization of murine T lymphocytes toward a type 2 cytokine profile was associated with a decrease in severity of experimental GVHD [5]. With respect to a potential molecular mechanism underpinning rG-CSF-mediated Th2 phenotypic transition, interaction of the cytokine with G-CSFR-expressing T cells induced an increase in mRNA expression and protein levels of the transcription factor GATA-3 (GATA3-binding protein) and a decrease in the level of expression of mRNA encoding the γ-subunit of the transcriptional regulator complex ISGF3 (interferon stimulated gene factor 3) [7,25,32]. GATA-3 is a transcription factor that plays critical roles in Th2 differentiation, Th2 cytokine production and selective growth of Th2 cells [33]. In addition, GATA-3 inhibits Th1 differentiation by blocking the IL-12 signaling pathway, neutralizing the function of RUNX3 (Runt-related transcription factor 3) and directly silencing the *ifng* gene [34]. Moreover, rG-CSF may also limit IFNγ signaling in T cells by suppressing expression of the gene encoding the ISGF3 subunit/p48 in cluster of differentiation (CD)4+ donor T cells [32].

### 4.3. T Helper 17 Cells

A gene profiling study of purified T cells isolated from the blood of rG-CSF-treated peripheral stem cell donors largely confirmed the aforementioned findings by revealing that genes related to Th2 cell-mediated immunity were upregulated, while those associated with Th1 cell function, including cytotoxicity, antigen presentation and GVHD, were downregulated [35]. In addition, overexpression of genes related to T regulatory cell (Treg) differentiation, as well as those that suppress Th17 phenotypic transition, were detected [35]. Although controversial [7], it is noteworthy that immunophenotyping procedures revealed that rG-CSF-exposed stem cell donors had reduced levels of T cells with a Th17 phenotype (CD4+IL-17+CCR6+IL-23R+) but increased expression levels of CD4+CD25^high^CD45RO+ T cells typical of Tregs [35]. Moreover, findings originating from the same study revealed that levels of mRNA encoding the Th17-specific transcription factor *RORγt* were significantly decreased in T cells isolated from G-CSF-mobilized peripheral blood stem cell harvests [35]. Further, also from a mechanistic perspective, another study demonstrated that rG-CSF directly modulated CD4+ T cell responses via upregulation of expression of the protein suppressor of cytokine signaling-3 (SOCS3), resulting in attenuation of Th17-mediated aGVHD [36]. In contrast, however, others have reported that utilization of rG-CSF to mobilize stem cells is associated with increased numbers of Th17 cells in the circulation that may exacerbate GVHD [37,38]. Clearly additional research is required to resolve these discrepancies [7,39].

### 4.4. T Regulatory Cells

As alluded to above, the mechanisms of rG-CSF-induced immune tolerance also include maturation of bone marrow-derived CD4+CD25+Foxp3+ Tregs that produce the immunosuppressive cytokines IL-10 and transforming growth factor-beta (TGFβ) [35]. High donor Treg content is associated with a low risk for the development of GVHD following allogeneic stem cell transplantation [40]. In this context, an early report by Rutella et al. (2002) [41] demonstrated that highly purified CD4+ T cells from healthy donors receiving rG-CSF acquired the functional properties of Treg type 1 (Tr1) cells that secreted high amounts of IL-10 and moderate amounts of TGFβ following activation with alloantigens in the absence of significant release of IL-2 and IL-4. These cells had a low proliferative capacity and mediated active suppression of antigen-driven proliferation of bystander T cells, seemingly by an IL-10/TGFβ-dependent mechanism [41,42]. These effects were consistent with observations derived from a murine model that revealed protection against development of GVHD following pre-treatment of the donors with pegylated rG-CSF that was dependent on the enhanced generation of IL-10-producing Tregs [43]. In addition, animal studies showed that administration of rG-CSF promoted the systemic expansion of natural (n) CD4+ CD25+ Tregs, depletion of which significantly exacerbated GVHD [44]. Importantly, however, depletion of Tregs did not completely negate the immunosuppressive effects of rG-CSF, consistent with the regulatory effects of rG-CSF on other types of immune cells [44]. In this context, it is noteworthy that rG-CSF upregulated several genes or gene families implicated in nTreg survival, including Pias3, an inhibitor of activated STAT3 (an established suppressor of nTreg stability), thereby enhancing nTreg stability and survival [7,44]. Furthermore, rG-CSF has been shown to reduce the expression of the chemokine CXCL12 in bone marrow, thereby inducing the homeostatic trafficking of CXCR4-expressing Tregs from human bone marrow to the periphery [45]. Apart from contributing to a protective effect in aGVHD, rG-CSF-induced Tregs have also been reported to protect against atherosclerosis, lupus nephritis and diabetes in murine studies [46,47,48].

### 4.5. CD8+ T Cells

Recombinant G-CSF exerts direct, suppressive effects on cytotoxic effector CD8^+^ T cells, resulting in partial attenuation of T cell function [49]. The effects of rG-CSF on CD8^+^ T cell function were investigated following antigen-dependent and -independent stimulation in vitro. In both cases, exposure to rG-CSF resulted in: (i) decreased secretion of IFNγ and granzyme B; (ii) reduced surface expression of the activation markers CD25, CD38, CD69 and CD137, as well as human leucocyte antigen D-related (HLA-DR); (iii) a decrease in the expression of microRNA-155, which is a key regulator of effector CD8+ T cells; and (iv) a reduction in the activation of T cell receptor (TCR)-dependent and -independent signaling pathways, as reflected by the phosphorylation of ERK1/2, Lck (lymphocyte-specific protein tyrosine kinase) and the CD3-ζ chain (CD247) [49]. All of these observations demonstrate that rG-GSF also directly suppresses cytotoxic CD8+ T lymphocyte function.

## 5. Effects of rG-CSF on Cells of the Innate Immune System

### 5.1. Dendritic Cells

Dendritic cells (DCs) are professional antigen-presenting cells that activate naïve T helper cells. T helper 1- and Th2-promoting DC lineages, known as DC1 and DC2, respectively, have been described [7]. In this context, administration of rG-CSF to healthy human donors resulted in systemic mobilization of large numbers of type 2 DCs, as well as CD4+ T cells expressing the Th2 phenotype [50,51]. Another study reported that administration of rG-CSF to mice resulted in induction of DCs that produced low levels of IL-12 [52]. Although not distinguishing between DC1 and DC2 cells, the authors speculated that their findings were consistent with reduced numbers of the former cell type that are critical for the differentiation of Th1 cells [52].

In addition, isolated human monocytes cultured with autologous serum derived from rG-CSF- treated healthy donors, which contained high levels of IL-10 and IFNα, differentiated into tolerogenic DCs in vitro that exhibited diminished IL-12p70 release and poor allostimulatory capacity, as well as induction of Tregs [53]. It has also been shown that human monocytes differentiated with rG-CSF and IL-4 acquire a DC-like morphology that is associated with spontaneous release of IL-10, leading to induction of anergy in naïve T cells in vitro [54].

The aforementioned findings appear to support the contention that transplantation of rG-CSF-augmented peripheral blood stem cells does not result in overwhelming aGVHD because the stem cell infusions contain predominantly Th2-inducing DCs [50].

### 5.2. Monocytes

As mentioned above, several earlier studies have indicated that the inhibitory effects of rG-CSF on T cell proliferation and cytokine responses were mediated indirectly by modulation of monocyte function [28,55]. In this context, rG-CSF pre-treatment of both a monocytic cell line (NOMO-1) and isolated human blood monocytes resulted in significant attenuation of LPS-induced secretion of the pro-inflammatory cytokines TNFα and IL-12 while augmenting production of the anti-inflammatory cytokine IL-10 [56]. In addition, the antigen-presenting function of rG-CSF-treated human monocytes was also found to be impaired [57]. A more recent publication reported that administration of rG-CSF to both humans and mice resulted in mobilization of a subset of immunosuppressive CD34+ cells that exhibited the features of mature monocytes [58]. In response to IFNγ released by activated T cells, these CD34+ cells induced allogeneic T cell death, resulting in expansion of Tregs and immunosuppression [58]. Moreover, and somewhat predictably, the presence of CD34+ monocytes in peripheral blood correlated inversely with the incidence of aGVHD in humans [58].

### 5.3. Myeloid-Derived Suppressor Cells

Myeloid-derived suppressor cells (MDSCs) are a heterogeneous group of myeloid cells of monocytic and granulocytic origin that suppress both innate and adaptive immune responses and undergo expansion during cancer, infection and inflammatory diseases [59]. It has been reported that rG-CSF-induced immune tolerance may be mediated by various MDSC subsets and that these are important regulators of the development of aGVHD in allogeneic hematopoietic stem cell transplantation [60]. In this context, expansion of myeloid cells in the peripheral blood of rG-CSF-treated human donors resulted in increased numbers of MDSC subtypes of both monocytic and granulocytic origin that significantly regulated alloreactive T cell responses in vitro [61]. In addition, a recent study revealed the existence of an unidentified, immature cell population of monocytic origin defined by the cell surface expression of HLA-DR^−/low^CD33^+^CD16^−^ in healthy donors that appeared following administration of rG-CSF [62]. This early MDSC-like population suppressed T cell proliferation in a TGFβ-dependent manner, skewed the T helper cell balance from a Th1 to Th2 predominance and promoted generation of Tregs [62]. Importantly, adoptive transfer of these cells prevented development of aGVHD in a humanized mouse model [62]. These findings are in agreement with those of earlier studies that identified the presence of immunosuppressive monocytic MDSCs that predicted the risk of aGVHD following infusion of rG-CSF-expanded peripheral blood stem cells [63,64].

### 5.4. Natural Killer Cells

Natural killer (NK) cells isolated from the blood of human rG-CSF-expanded stem cell donors, as well as following exposure to the cytokine in vitro, were found to be dysfunctional with respect to downregulation of expression of various activating receptors [NKp44, NKp46, NKGD2 (natural killer receptor group 2, member D)], as well as the inhibitory killer immunoglobulin-like receptors (KIRs) KIR2DL1 and 2 [65]. Additional abnormalities included a reduction in granzyme B levels that was associated with decreased cytotoxic/lytic capacity of NK cells both in vitro and in vivo [65,66,67], as well as decreased production of pro-inflammatory cytokines (IFNγ, TNFα, GM-CSF, IL-6, IL-8) and proliferative capacity [65]. More recently, Yu et al., who explored the effects of rG-CSF on NK cell subpopulations, found that administration of the growth factor not only decreased the percentage of circulating and bone marrow NK cells, but also altered the balance of NK subpopulations, resulting in a high ratio of CD56^bri^ to CD56^dim^ subsets in the setting of low levels of NK1 cells (IFNγ-secreting NK cells) [68].

### 5.5. Platelets

Administration of rG-CSF to humans prior to autologous stem cell transplantation is associated with decreased platelet counts [69]. In this context, experimental administration of rG-CSF to mice was found to induce thrombocytopenia by interfering with the differentiation of hematopoietic precursors into megakaryocytes [70]. Although this activity of rG-CSF may have implications for impairment of anti-viral and antimicrobial immunity mediated by both megakaryocytes [71] and platelets [72], the relevance, if any, to the clinical applications of rG-CSF remains to be established.

The immunomodulatory effects of rG-CSF, which presumably mimic those of endogenously generated G-CSF, are summarized in Table 1.

## 6. The Role of Endogenous G-CSF in the Pathogenesis of Cancer Progression and Invasion

Various types of advanced, solid malignancies produce G-CSF and also express its receptor, enabling autocrine proliferation of tumor cells, as well as conferring a strategy to intensify the immunosuppressive milieu of the tumor microenvironment (TME) via recruitment of immature and mature neutrophils that function as MDSCs [73]. Approximately 10% of patients with advanced, often metastatic, solid malignancies exhibit tumor-related leukocytosis, including, but not limited to, those of the lung, gastrointestinal tract, pancreas, breast and bladder [74]. In these settings, tumor-derived G-CSF induces accelerated myelopoiesis that results in a moderate and, in some instances, a profound leukocytosis that is associated with increased numbers of immature myeloid cells. These cells are found in the bone marrow, blood and spleen and their presence is predictive of a poor prognosis [10,73,74,75,76,77,78,79].

Particularly high levels of expression (~90%) of the G-CSFR have been reported in human gastric and colon cancers, a setting in which G-CSF originates not only from tumor cells per se, but also from stromal myofibroblasts/fibroblasts in the tumor microenvironment (TME) [80]. In the case of breast cancer, systemic levels of G-CSF are significantly increased in patients with advanced but not early disease, being highest in those with aggressive, invasive N3 tumors [81], with expression of the G-CSFR detected in 71% of patients with invasive ductal adenocarcinomas (stage T1-2 NOMO) [82]. In these and other types of advanced breast cancers such as triple negative breast cancer [83], as well as the high-risk luminal A dominant breast cancer subtype (C3) [84], high-level expression of G-CSF in biopsy specimens is associated with poor overall survival (OS) and invasive potential.

## 7. Mechanisms by Which G-CSF Promotes Tumor Progression and Invasion

Enhancement of tumor cell proliferation and immunosuppression represent the predominant mechanisms of G-CSF-driven tumor progression, while G-CSF-mediated pro-metastatic activity is achieved via several mechanisms that include augmentation of release of pro-angiogenic factors by various cell types in the TME, as well as via the involvement of neutrophil extracellular traps (NETs).

### 7.1. Tumor Cell Proliferation

The mechanisms by which G-CSF drives tumor progression vary according to the type of malignancy. For example, in the case of aggressive tumors with high-level expression of G-CSF/G-CSFR, such as breast cancer, gastric and colon cancers, squamous cell cancers of the head and neck and neuroblastoma, data derived from pre-clinical in vitro studies and animal models of tumorigenesis, as well as from histological/genotypic analysis of biopsy specimens from patients, have revealed the existence of autocrine mechanisms of tumor proliferation [10,80,85,86,87]. Intracellular signaling pathways involved in G-CSF/G-CSFR-activated tumor cell proliferation were, however, found to vary somewhat between tumor types. These mechanisms included the JAK/STAT pathway in the case of neuroblastoma cell lines [86,87] and the ERK1/2 and p90 ribosomal S6 kinase 1 pathways in gastric and colorectal cancer cell lines [80].

Alternative mechanisms, independent of G-CSFR-driven autocrine tumor cell proliferation, appear to promote G-CSF-mediated tumor progression in other types of malignancies such as experimental metastatic bone tumors [88] and Ewing sarcoma (ES) [89]. In the case of the former, a murine bone metastasis model was used that involved intra-tibial injection of a murine melanoma or a murine breast cancer cell line. Following subcutaneous administration of rG-CSF or vehicle (control) for eight days, tumor growth was assessed on the ninth day. The authors of the study observed that administration of rG-CSF resulted in significantly increased tumor growth that was dependent on the secretion of the pro-osteoclastogenic factor osteoprotegerin by osteoclasts, indicating that G-CSF-mediated osteoclastogenesis may enhance tumor growth in bone [88]. The relevance, if any, of these observations to the potential risk of short-term administration of rG-CSF in the clinical setting of chemotherapy-induced neutropenia in cancer patients has not, however, been established.

With respect to ES, analysis of biopsy material from 83 patients with this vascular malignancy revealed expression of G-CSF and G-CSFR protein and mRNA in all biopsy specimens, as well as in four ES cell lines [89]. Treatment of ES cell lines with rG-CSF in vitro failed to trigger proliferation of the cells. However, in a murine model of experimental tumorigenesis, administration of rG-CSF to nude mice for five days prior to injection of an ES cell line (TC/7-1) and continued administration of the growth factor for a further 14 day period resulted in significantly increased tumor growth relative to that of rG-CSF-untreated, control mice [89]. Although somewhat contentious [90], the authors speculate that the pro-tumorigenic effects of rG-CSF in this setting are linked to the release of progenitor cells from the bone marrow that promote tumor vascular expansion and growth [85]. Again, however, the clinical relevance of this proposed mechanism of endogenous G-CSF-associated tumorigenesis, if any, remains to be established.

### 7.2. Induction of Myeloid-Derived Suppressor Cells (MDSCs)

Notwithstanding the myelopoiesis-independent mechanisms of G-CSF-mediated immunosuppression mentioned in an earlier section of this review, proliferation and mobilization of MDSCs, specifically those of granulocytic leukocyte origin, represent the most probable mechanisms by which tumor-derived G-CSF promotes tumor progression [91]. In this setting, G-CSF operates in partnership with tumor-derived neutrophil chemoattractants, most prominently the chemokines CXCL5 (also known as epithelial neutrophil-activating peptide-78; ENA-78) and CXCL8 (IL-8) [92,93]. The resultant accumulation of MDSCs in the TME is conducive to the establishment of a highly immunosuppressive milieu [91]. In this setting, MDSCs are exposed to cytokines that promote differentiation of these cells and augmentation of their suppressive effects on the anti-tumor activities of tumor-infiltrating lymphocytes (TILs). In addition to G-CSF, cytokines encountered by MDSCs in the TME include most prominently TGFβ1 derived from various cell types including tumor cells per se, tumor-associated macrophages (TAMs) of the M2 phenotype, epithelial cells and fibroblasts [94,95,96,97]. In this setting, TGFβ1 is proteolytically cleaved to yield the bioactive protein by matrix metalloproteinase-9 (MMP-9) released by MDSCs [98].

Mechanisms utilized by MDSCs to suppress the functions of TILs have recently been described in detail elsewhere [99] and are summarized as follows:

- High-level production of anti-proliferative, cytotoxic reactive oxygen species (ROS) [99];
- Depletion of arginine and tryptophan via extracellular release of the enzymes arginase and indoleamine-2,3-dioxygenase [100,101];
- Expansion of Tregs via production of IL-10 [102];
- Interference with the recognition of tumor antigens via nitrosative modification of the T cell receptor for antigen [103];
- Physically impede the access of TILs to tumor cells in the TME [104];
- Via potentiation of neutrophil extracellular trap (NET) formation within the TME, which also obstructs the access of TILs to tumor cells [105].

In addition to promoting immunosuppression-associated tumor progression, the G-CSF/MDSC axis also contributes to tumor invasion and metastasis by driving NETosis within the TME.

## 8. Tumor Invasion/Metastasis

In addition to NETosis, other pro-invasive mechanisms involving the G-CSF/MDSC axis include MMP-9-driven epithelial-to-mesenchymal transition [106] and production of pro-angiogenic factors such as vascular endothelial growth factor (VEGF) and Bv8, as well as MMP-9 [106,107,108,109,110]. Furthermore, G-CSF released by tumor cells creates a pre-metastatic environment via mobilization of immature neutrophils from the bone marrow [111]. In this setting, these cells migrate to the metastatic site where they create an inflammatory, pro-metastatic milieu conducive to tumor cell invasion and proliferation, remodeling of the extracellular matrix and suppression of the anti-tumor immune response. In addition, the overexpression of G-CSF by various types of tumor cells has been shown to act as a “priming factor” that induces neutrophils to undergo NETosis [112,113].

### Role of Neutrophil Extracellular Traps in Tumor Invasion

NETosis is a highly regulated sequence of events that leads to cell death in response to various microbial and inflammatory stimuli. As a result of NETosis, neutrophils extrude their chromatin material leading to the formation of NETs [114]. Production of ROS by activated neutrophils leads to the disassembly of the nuclear envelope. This is followed by decondensation of the chromatin which is driven via activation of myeloperoxidase (MPO) [115], neutrophil elastase (NE) [116] and protein arginine deiminase type 4 (PAD4) [117]. The released chromatin, in turn, is “decorated” with various granular components including MPO, α-defensins, elastase, cathepsin G and lactoferrin, as well as histones [118,119]. NETs, therefore, aid in the immobilization and killing of invading microorganisms. However, the involvement of NETs in non-infectious inflammatory pathologies, including cancer, is now well recognized. NETs have been identified in patients with sarcoma [120], pancreatic cancer [121], breast cancer [122] and ovarian cancer [123]. In the context of cancer, NETs aid cancer cells in evading detection by cytotoxic TILs, or by forming a physical barrier between them [124]. They also play a role in metastasis through the trapping of tumor cells in vessels, thereby facilitating extravasation and tumor progression [125]. The progression of tumors by NETs is reported to be achieved through the promotion of thrombosis [113] and angiogenesis [126]. Due to the involvement of G-CSF, albeit indirect, in promoting NETosis in the TME, G-CSF has been proposed as a potential marker for individuals with cancer who are at risk of developing metastasis due to the formation of NETs [122].

## 9. Therapeutic Usage of Recombinant G-CSF

Chemotherapy-induced febrile neutropenia (FN) is a life-threatening after-effect of cancer treatment that poses a significant risk for development of infection-related morbidity and mortality, as well as substantial dose-limiting toxicity. The risk of infection increases with the severity and duration of neutropenia.

Patients developing grades 3 and 4 neutropenia (severe and life threatening, respectively) or FN during chemotherapy commonly require dose reductions and/or delayed administration of their chemotherapy, resulting in decreased relative dose intensity (RDI). Treatment outcomes may be compromised, mostly when the goal of treatment intent is either curative or to prolong survival. Accordingly, prophylactic use of rG-CSF is a therapeutic strategy for maintaining the RDI during treatment by decreasing the incidence of severe neutropenia and FN, thereby improving treatment outcomes and quality of life. Most commonly, rG-CSF is utilized to increase circulating neutrophil counts in patients facing severe and prolonged neutropenic episodes following chemotherapy. Additionally, as mentioned earlier, rG-CSF is used in patients and donors to mobilize peripheral blood stem cells for harvesting in the settings of autologous and allogeneic stem cell transplantation, respectively, as well as to support post-transplantation neutrophil recovery. The American Society of Clinical Oncology (ASCO), the National Comprehensive Cancer Network (NCCN) and the European Society for Medical Oncology (ESMO) have developed evidence-based guidelines defining the appropriate use of rG-CSF in cancer patients [127,128,129]. The main indications for the usage of rG-CSF are summarized in the following sections.

### 9.1. Indications for the Usage of rG-CSF

#### 9.1.1. Primary Prophylaxis

Primary prophylaxis is defined as the usage of rG-CSF in the first and subsequent cycles of chemotherapy. Most rG-CSF guidelines recommend primary prophylaxis for patients receiving chemotherapy regimens with an overall risk of FN that is greater than 20%. Patients receiving myelotoxic chemotherapy with curative or radical intent (including adjuvant/neoadjuvant settings) are included in this category. Additionally, primary prophylaxis should be considered for patients receiving myelotoxic chemotherapy with a documented FN incidence rate of 10–20% and who have one or more of the risk factors listed in Table 2 [129].

Primary prophylaxis is supported by the fact that approximately half of the neutropenic episodes occur during the first cycle of chemotherapy [130].

#### 9.1.2. Secondary Prophylaxis

Secondary prophylaxis is defined as the usage of rG-SCF in subsequent cycles after initial episodes of severe neutropenia and/or FN. This strategy may be considered if dose reduction or treatment delay may result in a decrease in disease-free or overall survival. Examples of types of malignancies in this category include adjuvant breast cancer, non-Hodgkin’s lymphoma, Hodgkin’s disease, testicular cancer and other germ cell tumors, as well as patients undergoing neoadjuvant chemotherapy with curative intention. For patients undergoing chemotherapy with palliative intent, dose modifications are considered a reasonable alternative.

#### 9.1.3. Supportive Therapy for Neutropenic Sepsis

Most guidelines do not recommend the use of rG-CSF for the treatment of patients with low-risk or uncomplicated FN, or afebrile neutropenia. Recombinant G-CSF is recommended for patients with severe or complicated FN as summarized in Table 3.

#### 9.1.4. Short-Acting vs. Long-Acting rG-CSF

Short-acting and long-acting rG-CSF are approved for the prophylaxis of FN [131]. Although observational, non-randomized studies have shown a greater efficacy of long-acting rG-CSF, most randomized studies have shown that there are no differences with respect to the duration of action between these formulations. The differences shown in observational studies may be a result of under-dosing patients with short-acting rG-CSFs as may occur in the real-world setting of general practice usage [132]. Importantly, biosimilar formulations of both forms of rG-CSF are available [133,134]. These represent a significant saving to healthcare systems [135].

#### 9.1.5. Other Clinical Recommendations for rG-CSFs

Additional clinical indications for the therapeutic usage of rG-CSF include idiosyncratic drug-induced neutropenia and agranulocytosis, congenital neutropenic syndromes and neutropenia-associated primary immunodeficiency disorders [136,137,138]. However, the clinical utility of rG-CSF in patients with myelodysplastic syndromes (MDS) remains unclear and controversial due to the effect of this agent on malignant clones. Some studies reported preferential stimulation of malignant clones, while others did not. There is a lack of significant evidence for the prophylactic use of rG-CSF in MDS, and most guidelines do not recommend its usage on a routine basis [139].

Colony-stimulating factors should be avoided in patients receiving concurrent chemotherapy and radiation therapy, particularly involving the mediastinum. In the absence of chemotherapy treatment, therapeutic use of CSFs may be considered in patients receiving radiation therapy alone if prolonged delays secondary to neutropenia are expected [121].

Hematopoietic syndrome is a clinical diagnosis given to people who present with equal or more than one new-onset cytopenia in the background of acute radiation exposure [140]. Recombinant G-CSF has been successfully used in this clinical setting. Additionally, a broad spectrum of agents has been investigated in the management of lethal acute radiation syndrome. These agents include immunomodulators, prostaglandins, inhibitors of prostaglandin synthesis, agonists of adenosine cell receptors, herbal extracts, flavonoids and vitamins [141].

Finally and reassuringly, long-term follow-up of healthy donors who had received rG-CSF in the setting of allogeneic stem cell transplantation failed to reveal a causal link with future development of hematological malignancies [142], underscoring the distinction between the clinical applications of rG-CSF and prolonged production of endogenous G-CSF by advanced malignancies. Nevertheless, the increasing prophylactic use of rG-CSF in the setting of medical oncology and hematology, together with the availability of more cost-effective biosimilars, clearly necessitates ongoing vigilance with respect to pro-tumorigenic potential [99,143,144,145].

## 10. Conclusions

Like many other cytokines, G-CSF is a rather enigmatic protein. On the one hand, controlled and appropriate production of G-CSF plays a critical role in anti-infective host defense, while on the other hand, inappropriate production by tumor cells appears to contribute to tumor growth and invasion. In the latter scenario, of which many clinicians may be unaware, the pro-tumorigenic effects are achieved via a dual mechanism of immunosuppression, seemingly by directing T cell polarization towards the Th2 and Treg phenotypes, and, most prominently, via induction of production of MDSCs. Given its key role in host defense, the most effective strategies to neutralize the pro-tumorigenic effects of endogenous G-CSF in the setting of adjunctive anti-cancer therapy are likely to involve pharmacologic targeting of the CXCR1 and CXCR2 chemokine receptors. The involvement of endogenously produced, tumor-derived G-CSF in tumorigenesis contrasts with the beneficial administration of rG-CSF in the prevention of chemotherapy-induced severe and life-threatening infection. Recent innovations in this setting include prophylactic use of rG-CSF, rather than delayed therapeutic administration following the onset of FN, thereby alleviating development of infection and treatment disruption. Although this strategy does not appear to pose the risk of development of hematological and other types of malignancies, ongoing vigilance is necessary, particularly in the context of increasing prophylactic utilization of rG-CSF and the availability of more cost-effective biosimilars. Future studies should be undertaken to monitor the emergence of MDSCs and/or alterations in the levels of their soluble, systemic mediators of immunosuppression in patients with cancer who receive prolonged/frequent administration of rG-CSF.

## Figures and Tables

**Table 1:** The immunomodulatory effects of recombinant granulocyte colony-stimulating factor (rG-CSF).

Cell Type Affected	Cell Response Affected	Reference
T cells		
T cell proliferative response	The proliferative response to allogeneic and mitogenic stimulation was profoundly reduced in patients and healthy stem cell donors treated with rG-CSF.	[22,23,24]
T helper 2 polarization	rG-CSF polarizes T cells from the Th1 to Th2 phenotype by skewing the cytokine secretion profile.	[5,28,29,30]
T regulatory cells	rG-CSF mobilization promoted expansion of Tregs; protective against aGVHD.	[34,42,43,44,47]
CD8+ T cells	rG-CSF directly reduces cytotoxic CD8+ T cell functionality.	[45]
Dendritic cells		
Th2 inducing DC2	Treatment with rG-CSF increased DC2 counts in peripheral blood. May drive a Th2 response.	[46]
Tolerogenic DCs	rG-CSF modulates monocyte differentiation towards tolerogenic DCs that cause anergy in naïve T cells.	[47,48]
Monocytes		
Monocyte-mediated production of proinflammatory cytokines	rG-CSF attenuated secretion of TNFα and IL-12 but caused an enhancement of IL-10 release.	[50]
CD34+ cells	rG-CSF mobilized stem cells contain immunosuppressive CD34+ cells (mature monocytes) that correlated inversely with incidence of aGVHD.	[51]
Myeloid suppressor cells		
MDSC (monocytic/promyelocytic)	Monocytic and promyelocytic MDSCs may contribute to rG-CSF-induced immune tolerance in allogeneic stem cell transplantation.	[53]
MDSC (monocytic/polymorphonuclear)	rG-CSF-mobilized donors contain mononuclear/polymorphonuclear MDSC subtypes that have the capacity to regulate alloreactive T cell responses in vitro.	[54]
Early MDSC-like population (HLA-DR^-/low^CD33^+^CD16^-^ immature monocytic cells)	These MDSCs were described in healthy donors after rG-CSF treatment. They suppressed T cell proliferation, facilitated Th2 differentiation and prevented aGVHD.	[55]
Monocytic MDSCs	Immunosuppressive cell population that could predict risk of aGVHD after transplantation of rG-CSF-mobilized peripheral blood stem cells.	[56,57]
Natural killer cells		
NK functionality	rG-CSF is an inhibitor of NK cell activity; decreases its cytotoxicity and production of pro-inflammatory cytokines by these cells.	[58,59,60]
NK subpopulations	rG-CSF applied in vivo not only decreased percentage of NK cells, but also modulated NK subpopulations leading to a high ratio of CD56^bri^ to CD56^dim^ subsets and low levels of NK1 cells (IFNγ-secreting NK cells).	[61]

**Table 2:** Risk factors associated with development of febrile neutropenia associated with an overall risk of 10–20%.

Patient Age > 65 Years
Poor performance status
Previous episodes of febrile neutropenia
Extensive prior treatment, including large radiation ports
Cytopenias due to bone marrow infiltration
Poor nutritional status
The presence of open wounds or active infections
Advanced cancer
Serious co-morbidities

**Table 3:** Complicated febrile neutropenia.

Profound febrile neutropenia with an absolute neutrophil count < 0.1 × 10^9^/L
Patients who are clinically unstable with hypotension and organ dysfunction
Established septic shock
Expected prolonged neutropenia of duration greater than 10 days
Persistent pyrexia despite appropriate antibiotics/antifungals
Presence of pneumonia
Proven or suspected invasive fungal infection
Being hospitalized at the time of the development of fever
